# Induction Mechanism of Ferroptosis, Necroptosis, and Pyroptosis: A Novel Therapeutic Target in Nervous System Diseases

**DOI:** 10.3390/ijms241210127

**Published:** 2023-06-14

**Authors:** Lu Tang, Sitong Liu, Shiwei Li, Ye Chen, Bingqing Xie, Jun Zhou

**Affiliations:** 1 Department of Anesthesiology, The Affiliated Hospital of Southwest Medical University, Luzhou 646000, China; 20210299120130@stu.swmu.edu.cn (L.T.); 20210299120517@stu.swmu.edu.cn (S.L.); 20210299120519@stu.swmu.edu.cn (S.L.); 2 Anesthesiology and Critical Care Medicine Key Laboratory of Luzhou, Southwest Medical University, Luzhou 646000, China; chenye0117@swmu.edu.cn; 3 Department of Traditional Chinese Medicine, The Affiliated Hospital of Southwest Medical University, Luzhou 646000, China; 4 Laboratory of Neurological Diseases and Brain Function, The Affiliated Hospital of Southwest Medical University, Luzhou 646000, China; bingqingxie0503@126.com; 5 Institute of Epigenetics and Brain Science, Southwest Medical University, Luzhou 646000, China

**Keywords:** ferroptosis, necroptosis, pyroptosis, nervous system disease, neurodegenerative disease, traumatic brain injury, stroke

## Abstract

In recent years, three emerging cell deaths, ferroptosis, necroptosis and pyroptosis, have gradually attracted everyone’s attention, and they also play an important role in the occurrence and development of various diseases. Ferroptosis is an idiographic iron-dependent form regulated cell death with the hallmark of accumulation of the intracellular reactive oxygen species (ROS). Necroptosis is a form of regulated necrotic cell death mediated by the receptor-interacting protein kinase 1(RIPK1) and receptor-interacting protein kinase 3RIPK3. Pyroptosis, also known as cell inflammatory necrosis, is a programmed cell necrosis mediated by Gasdermin D (GSDMD). It is manifested by the continuous swelling of the cells until the cell membrane ruptures, resulting in the release of the cell contents and the activation of a strong inflammatory response. Neurological disorders remain a clinical challenge and patients do not respond well to conventional treatments. Nerve cell death can aggravate the occurrence and development of neurological diseases. This article reviews the specific mechanisms of these three types of cell death and their relationship with neurological diseases and the evidence for the role of the three types of cell death in neurological diseases; understanding these pathways and their mechanisms is helpful for the treatment of neurological diseases.

## 1. Introduction

Cell death is common in a variety of organisms and is closely associated with a lot of diseases. In recent years, with the deepening of research on the mechanism of cell death, a variety of cell deaths have been defined [1]. Ferroptosis, necroptosis and pyroptosis are three types of non-apoptotic cell death that have been extensively studied [2]. At present, the great achievements of cell death in cancer have been reported, and the induction of cell death mechanisms other than apoptosis has become a new strategy for cancer treatment [3]. Of course, cell death is a double-edged sword, which can promote the occurrence of some diseases. In the nervous system, the normal growth and development of nerve cells is crucial to the nervous system. However, abnormal death of nervous system cells can lead to a series of nervous system diseases. Common nervous system diseases in clinic include neurodegenerative diseases, stroke, traumatic brain injury, etc. The etiology of the nervous system is multifactorial. With the research on nervous system diseases, it is found that their pathogenesis is closely related to cell death. We would like to explore the treatment of its disease by summarizing and comparing these cell death pathways. Understanding the regulation of cell death can help prevent and treat disease.

## 2. Ferroptosis

### 2.1. Definition

Ferroptosis was discovered in recent years; it is a new form of cell death. Cell death is usually accompanied by the accumulation of large amounts of iron, lipid peroxidation and ROS [4]. Ferroptosis occurs in a completely different process from other types of cell death [5]. Morphologically, ferroptosis is characterized by an intact cell membrane, normal nuclear morphology, atrophy of mitochondria, increased density of the mitochondrial membrane, and a reduced or disappeared density of the mitochondrial cristae [6]. Biochemically, ferroptosis is mainly due to the depletion of glutathione (GSH) in the cell and the inactivation of glutathione peroxidase 4 (GPX4) for lipid-peroxide reduction, resulting in an increased lipid peroxidation and lipid ROS [7] (Table 1).

### 2.2. Mechanism

#### 2.2.1. System Xc-/GPX4

**Figure 1:**
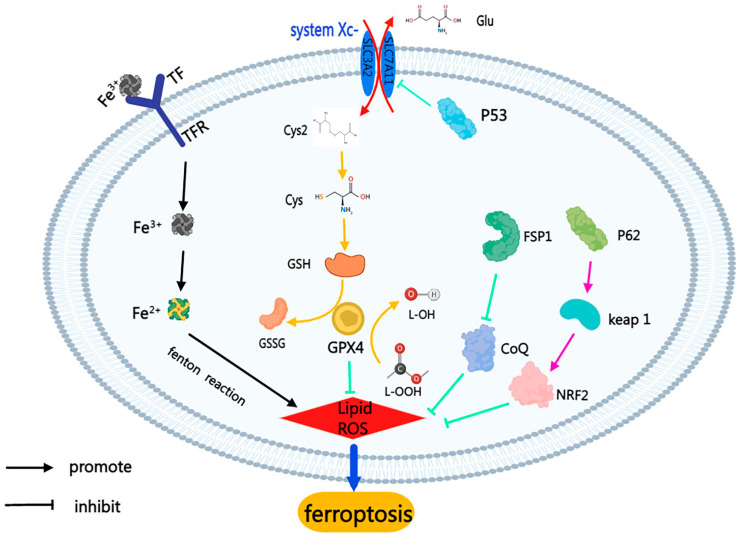
The mechanism of ferroptosis. The process of ferroptosis is usually accompanied by the accumulation of lipid peroxides and the disorder of iron metabolism. By inhibiting the System Xc-, it inhibits the transfer of cystine into cells to participate in the synthesis of GSH, leading to the occurrence of ferroptosis. Fe^3+^ enters cells and is reduced to Fe^2+^ in Fenton reaction, which produces a large amount of ROS. In addition, the promotion of lipid–peroxide production and disturbance of iron ion metabolism induce ferroptosis through other pathways.

Suppression of System Xc- to induce ferroptosis: System Xc- consists of SLC7A11 and SLC3A2, embedded on the surface of the cell membrane. Glutamate and cystine (Cys2) are exchanged inside and outside the cell via System Xc- [19]. The Cys2 transferred into the cell is further reduced to cysteine (Cys) and participates in GSH synthesis. The inhibitory System Xc- promotes ferroptosis by inhibiting cystine uptake [19,20] (Figure 1).

P53-induced ferroptosis is mediated by the inhibition of the SLC7A11 transcription, which inhibits cystine uptake by System Xc-, resulting in decreased GSH and inducing ferroptosis of the cells [21,22,23].

Deactivation of GPX4: GPX4 is an antioxidant enzyme and a restricted GSH-dependent enzyme [24]. GPX4 reduces the toxic lipid peroxides to their corresponding alcohols, weakening their toxicity. Any factor that inhibits GPX4 activity will result in the accumulation of lipid peroxides, which induces ferroptosis [20].

Depletion of GSH: Depriving cells of the essential GSH precursor Cys or blocking the function of the GSH-dependent enzyme GPX4 can induce ferroptosis [25].

#### 2.2.2. FSP1-COQ10 Pathway

Ferroptosis suppressor protein 1 (FSP1) can function as an oxidoreductase in the plasma membrane, reduce coenzyme Q10 (CoQ10), and prevent the propagation of lipid peroxides. Loss of FSP1 can lead to an increase in lipid peroxides, which can lead to ferroptosis [26] (Figure 1).

#### 2.2.3. P62-Keap1-NRF2 Pathway

p62 mediates Kelch-like ECH-associated protein 1 (Keap1) degradation, which dissociates nuclear factor erythroid 2-related factor (Nrf2) from Keap1 and the unbound Nrf2, to enter the nucleus to regulate downstream genes. Therefore, Nrf2 activation suppresses ferroptosis [27]. Nrf2 is an important antioxidant transcription factor, and p62-mediated Keap1 degradation contributes to Nrf2 activation during ferroptosis. Nrf2-regulated genes NQO1, HO1 and FTH1 confer a resistance to ferroptosis by altering iron metabolism and lipid peroxidation [27] (Figure 1).

#### 2.2.4. Iron Accumulation

Transferrin (Tf) and transferrin receptor (TfR) form the Tf-TfR complex [28]. The Tf-TfR complex transfers Fe^3+^ into cells, where it is reduced to Fe^2+^ [29]. Intracellular iron accumulation leads to massive ROS generation by the Fenton reaction to produce hydroxyl radicals. Both the disruption of iron homeostasis and increased levels of ferrous iron can lead to ferroptosis [30] (Figure 1).

## 3. Necroptosis

### The Definition and Mechanism of Necroptosis

**Figure 2:**
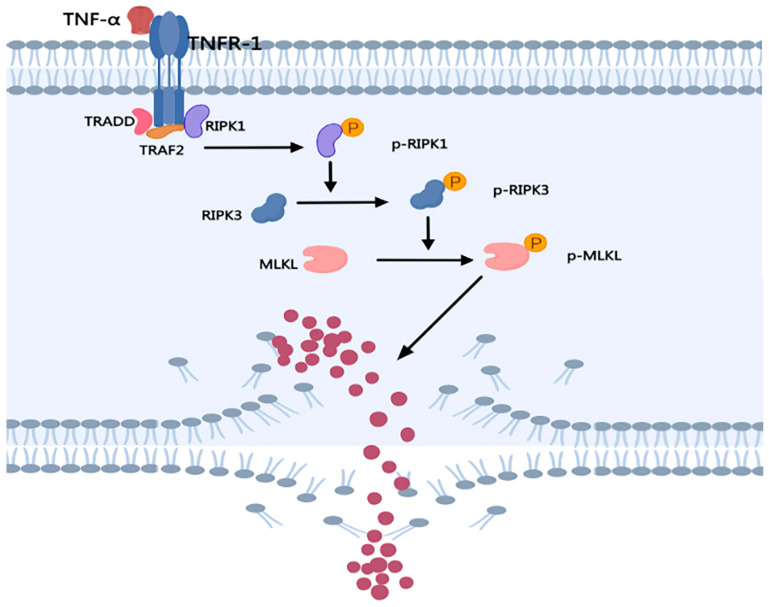
The mechanism of necroptosis. The death receptor TNFR binds to its ligand and forms a corresponding complex to activate RIPK1. After phosphorylation of RIPK1, RIPK3 is recruited and RIPK3 is phosphorylated and activated. RIPK3 acts on MLKL to phosphorylate, and the phosphorylated MLKL oligomerizes into a pore-like structure on the cell membrane, leading to rapid cell membrane disruption.

Necroptosis is different from apoptosis; it is a new cell death pathway [31], which can occur when the apoptotic pathway is blocked [32]. In 2008, RIPK1 was identified as a specific molecular target of necroptosis [33]. In 2009, RIPK3 was identified as a key regulator of necroptosis [34]. In 2012, MLKL was shown to be a key molecule in downstream signaling of necroptosis [35]. Morphologically, necroptosis includes organelle swelling and the loss of plasma membrane integrity. The breakdown of cell membranes leads to the release of cell contents, which results in an inflammatory response [8] (Table 1). In this specific mechanism: TNFα binds to TNF receptor 1 (TNFR1) on the cell membrane and recruits the protein to form a complex [9]; the complex includes TNFR-associated death domain (TRADD), RIPK1, TNFR-associated factor 2 (TRAF2), cellular inhibitor of apoptosis 1 (cIAP1), cylindromatosis (CYLD), and NF-κB essential modulator NEMO [9,10]. After phosphorylation of RIPK1, RIPK3 is recruited and RIPK3 is phosphorylated and activated. RIPK3 acts on MLKL to phosphorylate, and the phosphorylated MLKL oligomerizes into a pore-like structure on the cell membrane, leading to rapid cell membrane disruption [11,12,13] (Figure 2).

## 4. Pyroptosis

### The Definition and Mechanism of Pyroptosis

**Figure 3:**
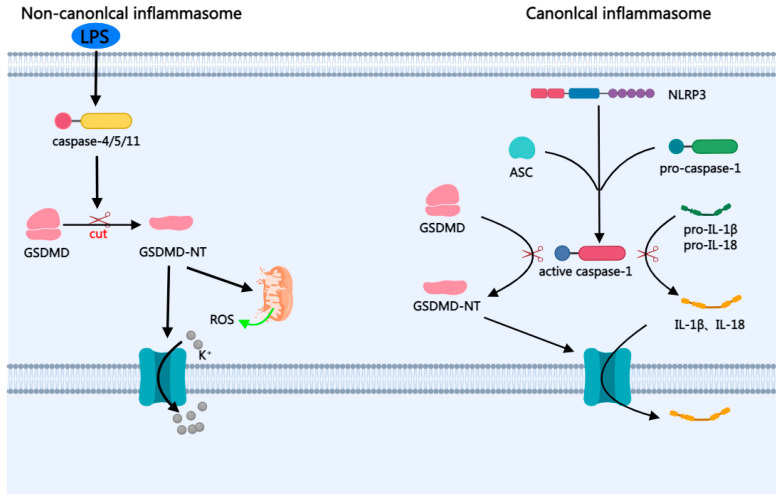
The mechanism of pyroptosis. Pyroptosis mainly includes two signaling pathways, the canonical inflammasome pathways and non-canonical inflammasome pathways. In canonical inflammasome pathways, various triggers activate recognition receptors (NLRP3, NLRP1, AIM2) to form inflammasome complexes. The complex leads to the recruitment of an adaptor protein apoptosis-associated ASC and the activation of caspase-1, which cleaves GSDMD into GSDMD-NT and GSDMD-CT. GSDMD-NT processes IL-1β and IL-18 into mature cytokine. GSDMD-NT mediates plasma membrane pore formation, thereby releasing mature IL-1β, IL-18, leading to pyroptosis. Non-canonical inflammasome pathways: LPS promotes oligomerization and activation of caspase-4/5/11, processes caspase into mature caspase, cleaves GSDMD into GSDMD-NT and GSDMD-CT, and GSDMD-NT mediates plasma membrane pore formation. Meanwhile, the cleavage of GSDMD triggers mitoROS and K+ efflux, hereby activating the downstream NLRP3 inflammasome.

The defining characteristic of pyroptosis is the inflammatory necrosis of cells. Pyroptosis is mediated by an inflammatory caspase, which activates a gasdermin protein translocation to the cell membrane to form pores leading to cell disruption [36] (Table 1). Pyroptosis mainly includes two signaling pathways, the canonical inflammasome pathways and non-canonical inflammasome pathways [14,15,16]. For canonical inflammasome pathways, various triggers activate recognition receptors (NLRP3, NLRP1, AIM2) to form inflammasome complexes [37]. The complex leads to the recruitment of an adaptor protein apoptosis-associated speck-like protein containing (ASC) and the activation of caspase-1, which cleaves gasdermin D (GSDMD) into gasdermin-D N-terminal domain (GSDMD-NT) and gasdermin-D C-terminal domain (GSDMD-CT). GSDMD-NT processes IL-1β and IL-18 into mature cytokine. GSDMD-NT mediates plasma membrane pore formation, thereby releasing mature IL-1β, IL-18, leading to pyroptosis [36,38]. Non-canonical inflammasome pathways: lipopolysaccharide (LPS) promotes oligomerization and activation of caspase-4/5/11, processes caspase into mature caspase, cleaves GSDMD into GSDMD-NT and GSDMD-CT, and GSDMD-NT mediates plasma membrane pore formation [39]. Meanwhile, the cleavage of GSDMD triggers mitochondrial ROS (mitoROS) and K+ efflux, hereby activating the downstream NLRP3 inflammasome [40,41,42,43] (Figure 3).

## 5. These Three Types of Cell Deaths Are Different from Apoptosis

Apoptosis is the first defined programmed cell death. With the advent of the concept of apoptosis, people pay more attention to a series of pathophysiological changes caused by programmed cell death. There are two main pathways of apoptosis: the mitochondrial pathway and death receptor pathway [44]. The mitochondrial pathway is caused by DNA damage or endoplasmic reticulum stress [45]. The death receptor pathway is initiated through the involvement of death-inducing receptors Fas and TNF-α receptor (TNFR) [46]. Apoptosis mainly involves the encapsulation of dying contents in apoptotic bodies, while both pyroptosis and necroptosis result in the rupture of the cell membrane by changing the permeability of the plasma membrane, resulting in the outflow of intracellular material [8,40,41,42,43,47]. Unlike the two types of cell death mentioned above, apoptosis does not cause inflammation and the breakdown of cell membranes [48]. The process of ferroptosis is usually accompanied by the accumulation of large amounts of iron, lipid peroxidation, and ROS [4]. This is also completely different from apoptosis(Table 1).

## 6. Association of Cell Death with Neurodegenerative Diseases

Neurodegenerative disease is associated with many factors, including inflammation, mitochondrial dysfunction, cell death, and more. Studies have shown that ferroptosis, pyroptosis, and necroptosis take part in neurodegenerative diseases [49,50]. However, it is unclear whether cell death plays a decisive or auxiliary role in the occurrence and development of neurodegenerative diseases, and how cell death regulates the nervous system, and by which cell death pathways improve the outcome of neurodegenerative diseases. The following summarizes the specific roles of ferroptosis, necroptosis and pyroptosis in neurodegenerative diseases.

### 6.1. Ferroptosis and Neurodegenerative Diseases

Ferroptosis is a common pathological condition in several neurodegenerative diseases, such as: Parkinson’s disease (PD), Alzheimer’s disease (AD), and Huntington’s disease (HD). Iron deposition in the brain can affect neurons and nerve cells [51] (Table 2).

#### 6.1.1. Ferroptosis and Parkinson’s Disease

The changes of iron content in the substantia nigra of PD may be related to the pathophysiology and treatment of the disease [74]. In earlier studies, it was found the glutathione pathway in PD is severely damaged, and the level of glutathione in the substantia nigra is reduced compared with normal people [52]. Ferrostatin-1(Fer-1) is an inhibitor of ferroptosis, which can inhibit ferroptosis in a variety of disease models such as PD, AD, and HD [75,76,77]. PD patients treated with iron chelators showed clinical and radiological improvement [53] because iron chelators protects neurons in PD from damage by ferroptosis [78]. In neurodegenerative degeneration, iron chelators and Fer-1 derivatives maybe potent drug candidates for pharmacological modulation of ferritin-signaling cascades [79].

#### 6.1.2. Ferroptosis and Alzheimer’s Disease

The brains of AD patients exhibit an enhanced expression of the iron storage proteins ferritin-heavy chain (FTH) and ferritin-light chain (FTL) [54]. Ferritin can increase oxidative stress through the Fenton reaction [80]. GSH is decreased in the hippocampus (HP) and frontal cortex (FC) of the brain of AD patients, and decreased GSH in these regions is associated with decreased cognitive function [55]. Hippocampal neuron GPX4 knockout (Gpx4BIKO) mice showed decreased spatial learning ability and memory, and a degeneration of hippocampal neurons [56]. Both iron imbalance and GSH reduction contribute to ferroptosis in AD patients. Inhibiting the inducing factors of ferroptosis maybe an effective treatment of AD.

#### 6.1.3. Ferroptosis and Huntington’s Disease

HD is also a neurodegenerative disorder, mainly in the striatum and cortex [81]. At present, the mechanism of cognitive and psychiatric symptoms in HD patients is not clear, but striatal dopaminergic dysregulation is thought to be a key factor in inducing the disease. A study finds early-stage HD patients have increased iron levels [57,82]. Subsequent further studies found that early iron increases in HD patients mainly occurred in the striatum and globus pallidus (GP) [57]. HD patients had lower GSH compared to healthy subjects [83,84]. However, another study showed significantly increased GSH content in mitochondria isolated from the cortex and striatum in HD patients [85]. One study showed that the activity of GPX in peripheral blood is reduced in HD patients [86], but another study showed that fibroblasts in HD patients did not differ between the GPX activity [87]. This difference may be caused by different tissue sources. The role of dysregulation of GSH and GPX in HD patients should not be ignored, but its contradictory results deserve further exploration [88]. Studies have shown that Nrf2 activation in HD patient-derived neural stem cells, which can inhibit ferroptosis. The mechanism is that Nrf2 activation can increase the expression of antioxidant proteins and reduce ROS levels in the brain. In addition to this, Nrf2 activation was able to suppress inflammatory responses in mouse microglia and astrocytes, the major cellular mediators of neuroinflammation, as well as in blood monocytes from HD patients [58]. Fer-1 and analogues are protective against cell death in a brain slice model of HD [75,89]. These results show that ferroptosis is closely related to neurodegenerative diseases. But there are a lot of ways to trigger ferroptosis, and it is not clear which pathway or if several pathways synergistically act through various diseases. Research on ferroptosis is still in its preliminary stages. Ferroptosis can be inhibited by inhibitors, thereby protecting cells and related organs. In neurodegenerative diseases, we are still limited to treating the symptoms rather than the disease. Important advances in the treatment of this disease can be made as the pathogenesis is more understood.

### 6.2. Necroptosis and Neurodegenerative Diseases

Necroptosis is a programmed cell death pathway that regulates RIPK1 and RIPK3 following activation of death receptor signaling, resulting in MLKL oligomerization into pore-like structures at the plasma membrane [11,12,13]. Recent studies have shown that necroptosis is related to the pathogenesis of a variety of neurodegenerative diseases(Table 2).

#### 6.2.1. Necroptosis and Parkinson’s Disease

PD disease is characterized by the degeneration of dopaminergic neurons in the substantia nigra. It has been reported that RIPK1 activation induced by inhibition of miR-425 has been implicated in neurodegeneration of dopaminergic neurons [59]. Furthermore, the levels of RIPK1, RIPK3 and MLKL proteins associated with necroptosis in the substantia nigra of PD patients were significantly increased [59,60,90]. In the PD model, an early increase in the interaction between RIPK1 and pMLKL in the striatum was observed, suggesting the formation of necrosome complexes [61]. Inhibition of RIPK1 by using a RIPK1 inhibitor (Nec-1) can protect dopaminergic neurons in a PD model [60].

#### 6.2.2. Necroptosis and Alzheimer’s Disease

It has been demonstrated that necroptosis is activated in the brains of AD patients, as evidenced by the increased expression levels of RIPK1 and MLKL, two key proteins of necroptosis, in the brain of AD patients [91]. Necroptosis is definitely activated in the brains of AD patients [62,91], but the mechanism by which it is activated is still unclear. AD is due to progressive neurodegeneration, leading to cognitive impairment, memory loss, and dementia. The neuropathological features of AD include neuronal deposits of hyperphosphorylated tau(pTau) protein and accumulation of both intracellular and extracellular aggregates of amyloid-β(Aβ). Studies have shown that Aβ oligomer-mediated stimulation of microglia in AD contributes to neuronal necroptosis activation and neurodegeneration [92]. Reduction of O-linked β-N-acetylglucosaminylation in AD patients has been implicated as a trigger of necroptosis [63].The activation of TNFR1-mediated necroptosis in hippocampal neurons of AD patients after death, and TNF-TNFR1 interaction, may be the main mechanism driving neurodegeneration in AD [64]. It was found that the inhibition of necroptosis (i.e., Nec-1) not only ameliorated neurodegeneration in mice, but also helped to relieve the symptoms of AD and reduce Aβ [65,93,94,95]. At present, the mechanism of necroptosis activation in AD is not perfect, but the detection of key proteins of necroptosis in the brain of postmortem AD patients proved that necroptosis indeed exists in AD. Additionally, Nec-1 alleviating AD-related symptoms also indicates that there is indeed an activation of necroptosis signaling.

#### 6.2.3. Necroptosis and Huntington’s Disease

The delay of disease onset by Nec-1 in R6/2 transgenic mouse of HD further confirms the involvement of RIP1 signaling in disease pathogenesis and can be considered as a potential therapeutic approach to improve symptoms in HD patients [66].

This study proved that the occurrence of HD is related to the activation of necroptosis; however, the research in this field is limited, and there are no reports on necroptosis proteins in HD patient samples.

Myriad evidence shows that necroptosis plays a crucial role in neurodegenerative diseases, but there are few related studies on the direct connection between necroptosis and neurodegenerative diseases. It mainly inhibits necroptosis by inhibiting the expression of key proteins. Inhibitors have a certain positive effect on the outcome of the disease. There are still many problems to be solved.

### 6.3. Pyroptosis and Neurodegenerative Diseases

Inflammasomes can cause inflammation and pyroptosis. Among various types of inflammasomes, NLRP3 inflammasome is closely related to neurodegenerative diseases. The inflammasome is a key mediator involved between CNS inflammation and cell death [96]. Pyroptosis may be one of the causes of neurodegenerative diseases(Table 2).

#### 6.3.1. Pyroptosis and Parkinson’s Disease

The activation of microglia is related to the pathogenesis of PD, and microglia activation may directly release of various substances (IL-1β, TNF-α, IL-6), NO, PGE (2) and superoxide, which directly lead to neuronal toxicity [97]. Several studies have suggested that neuroinflammation perhaps involved neuronal degeneration through the production of harmful proinflammatory cytokines [98]. Pyroptosis can promote the inflammatory response by promoting the secretion of inflammatory factors and the activation of inflammasomes, leading to neuronal degeneration in PD [67]. α-synuclein is one of the most studied proteins related to the pathogenesis of PD [99]. The linear correlation between NLRP3, the downstream protein of pyroptosis, and α-synuclein in serum of PD patients suggests that pyroptosis may be related to the pathogenesis of PD [68]. Current research lacks evidence that the gasdermin protein is directly activated in PD patients, but the impact of inflammation on PD disease has attracted attention, and multiple studies have shown that inhibiting inflammation can be beneficial for PD patients.

#### 6.3.2. Pyroptosis and Alzheimer’s Disease

Amyloid β-protein (Aβ) is closely related to the pathogenesis of AD [100]. Aβ aggregates can activate the NLRP3 inflammatory pathway, which promotes maturation of caspase-1. NLRP3 and caspase-1 are activated to promote the release of inflammatory factors and promote the polarization of microglia to a pro-inflammatory phenotype [16,61,91]. Intervention of pyroptosis can attenuate neuroinflammation in AD, reduce nerve cell damage and improve cognitive impairment in AD [70]. Some studies have confirmed that the levels of IL-1β and caspase-1 in the cerebrospinal fluid of AD patients are increased [69,101]. In addition, recent studies have shown that the expression of GSDMD in their cerebrospinal fluid is also significantly increased, and the increase of GSDMD expression indicates the occurrence of pyroptosis in AD [71]. However, there are few relevant studies at present, and more data support is still needed.

#### 6.3.3. Pyroptosis and Huntington’s Disease

In a mouse model of HD, an increased expression of caspase-1, caspase-8, and NLRP3 was discovered in striatal neurons [72]. At present, the study of pyroptosis in HD is still in the initial stage, and there is no evidence that the gasdermin protein is directly activated in HD patients, but the inhibition of inflammatory pathways is helpful for the prognosis of HD. The use of a selective inhibitor of NLRP3 inflammasome in transgenic mouse of HD has been shown to reduce IL-1β and reactive oxygen species production, as well as reduce neuronal toxicity, reduce neuroinflammation, prolong life span and improve motor dysfunction [73]. It provides a certain direction for the treatment of HD patients in the future, leading to a series of pathological changes

## 7. Association of Cell Death with Traumatic Brain Injury

Traumatic brain injury(TBI) is caused by external physical forces, such as blows, penetrations, or explosions, leading to a range of pathological changes in normal brain function [102]. It mainly includes primary injury and secondary injury. Mechanisms of primary injury include disruption of the blood–brain barrier, damage to axonal fibers, and cell death [103]. Mechanisms that lead to secondary damage include oxidative stress, lipid peroxidation, and inflammation [104].

### 7.1. Ferroptosis and Traumatic Brain Injury

According to some studies, there is a direct or indirect association between ferroptosis and the pathological changes of traumatic brain injury. Ferroptosis has been demonstrated in animal models of TBI. Iron accumulation in the thalamus of TBI model mice was observed by MRI [105]. Furthermore, it has been shown that the GPX4 protein decreased in the early stage after TBI, and returned to normal level by 7 days after injury [106]. In addition to this, GSH is reduced or even depleted after TBI [107]. Consistent with the above findings, GSH reduction was observed in the serum of patients with clinically mild TBI [108]. Moreover, Fer-1 can reduce neuronal cell death and improve cognitive and motor dysfunction caused by brain injury [109]. Additionally, in the experiment and clinical observation, various lipid oxidation indexes were increased in the brain tissue or cerebrospinal fluid of TBI patients [110]. System Xc- is regulated by glutamate in ferroptosis. After TBI, the extracellular glutamate concentration increases significantly and system XC- is inhibited, which induces ferroptosis [111]. Glutamate release after TBI is responsible for excitotoxicity after brain injury, leading to neuronal damage [112]. The reduction of glutamate release or inhibition of glutamate receptor activation has been shown to be neuroprotective after TBI [113]. In the TBI model, ferroptosis is induced through multiple pathways, and inhibition of ferroptosis is beneficial to the prognosis of TBI patients(Table 3).

### 7.2. Necroptosis and Traumatic Brain Injury

The increased expression of necroptosis signaling molecules RIPK1 and RIPK3 was found after TBI, and necroptosis is thought to be involved in TBI disease [120]. The values of RIPK3 and p-MLKL in the TBI mouse model peaked at an early stage, so it can be concluded that necroptosis occurs at an early stage of TBI. The inhibition of necroptosis can improve its prognosis [114]. However, some researchers have found that necroptosis signaling molecules RIPK1 and RIPK3 are mainly involved in chronic neuronal injury after TBI. RIPK3-global knockout animals, as well as neuronal RIPK1-deficient mice, were significantly protected against chronic brain injury, whereas no protective effect was observed during the acute phase of TBI, indicating that necroptosis mainly mediates chronic traumatic brain injury [115]. This contradicts the conclusion that necroptosis occurs in the early stages of TBI, but for TBI patients, acute injury is often fatal, so it is more meaningful to alleviate acute injury by inhibiting necroptosis. In addition to animal models, it has also been validated in human models, and compared with normal brain tissue, whereas TBI patient tissue shows morphological features of necroptosis and elevated levels of RIPK1, RIPK3, and MLKL proteins [121]. The inhibition of RIPK3 may reduce TBI injury by inhibiting inflammation and oxidative stress [116]. At present, the research of necroptosis in a TBI model is still in its infancy, and some studies are still controversial and contradictory, and further study on the causal relationship between the two is needed (Table 3).

### 7.3. Pyroptosis and Traumatic Brain Injury

More and more evidence has proved that pyroptosis is closely related to the pathogenesis of TBI. TBI induces activation of the NLRP3-inflammasome, resulting in increased ASC and caspase-1 expression and maturation of IL-1β and IL-18 [122,123]. Ac-FLTD-CMK inhibits pyroptosis by inhibiting caspase1/4/5 and reducing the secretion of IL-1β [124]. At the same time, the use of Ac-FLTD-CMK pyroptosis inhibitor can significantly downregulate the expression of caspase-1, GSDMD N-terminal, IL-1β and IL-18, reduce neuronal death, and improve neurobehavioral function. This showed that Ac-FLTD-CMK inhibited the pyroptotic process and protected mice from TBI [117]. Both caspase-1 inhibitors and NLRP3 inhibitors can exert neuroprotective effects after TBI [118,119,125]. Various inhibitors of the pyroptotic pathway can play a certain protective role, providing potential targets for the clinical treatment of TBI (Table 3).

## 8. Association of Cell Death with Stroke

Stroke mainly includes ischemic stroke and cerebral hemorrhage [126]. Ischemic stroke is a sudden neurological deficit caused by an interruption of cerebral blood flow, and has a high mortality and disability rate. Some traditional risk factors, such as diabetes and hypertension, are associated with the disease, but some risk factors for ischemic stroke remain unexplained [127]. Cerebral hemorrhage refers to a primary, spontaneous, nontraumatic hemorrhage that occurs in the brain parenchyma, including intracerebral hemorrhage (ICH) and subarachnoid hemorrhage (SAH). The main risk factors are hypertension, cerebral amyloid angiopathy, and the use of anticoagulants [128]. In recent years, with the in-depth study of stroke and cell death, it has been found that there is a close relationship between them.

### 8.1. Ferroptosis and Stroke

With the growth of social economy, the incidence of ischemic stroke is gradually increasing, and its poor prognosis is also a big problem for clinicians, so it is very important to find its treatment targets and improve the prognosis of patients. Palmer et al. found that an accumulation of iron in the cortex of hypoxic ischemic neonatal rats [129]. Several clinical studies have also shown that systemic iron loading increases brain damage in ischemic stroke and leads to a poor prognosis [130,131]. Free iron can exert its neurotoxic effects by generating hydroxyl radicals under hypoxic conditions. In an ischemic stroke model, iron depletion or chelation reduces cerebral edema and metabolic exhaustion, and inhibits iron-dependent lipid peroxidation, thereby attenuating neuronal damage [132]. The causal relationship between the two is currently unclear, as to whether ischemic stroke leads to iron accumulation and aggravates its damage, or if iron accumulation is a risk factor for ischemic stroke. Further research is needed to demonstrate its causal relationship and provide targets for the prevention and subsequent treatment of its disease.

Hemoglobin is released from lysed erythrocytes after ICH; there is evidence that released hemoglobin aggravates neuronal damage, and hemoglobin inhibits GPX activity and leads to the accumulation of lipid ROS [133]. GPX4 was decreased in the brain after ICH was compared with the Sham group, and reached a minimum level at 24 h, while the increase of GPX4 level alleviated brain edema, blood–brain barrier (BBB) damage, neuronal dysfunction, oxidative stress and inflammation after ICH. The inhibition of ferroptosis or upregulation of GPX4, ameliorates ICH-induced brain injury [134]. These all suggest that ferroptosis aggravates the progression of ICH disease, and the inhibition of ferroptosis may be a potential target for future treatment of this disease(Table 4).

### 8.2. Necroptosis and Stroke

Endothelial cell (EC) death can lead to vascular injury and BBB disruption, and BBB disruption after ischemic stroke is a catastrophic event [142]. Ischemic injury-induced necroptosis occurs mainly in brain ECs and neurons [143]. Cerebral ischemia induced phosphorylation of RIPK1 and increased the expression levels of RIPK3 and MLKL in the brain. Treatment with Nec-1 significantly inhibited these changes, thereby protecting neurological function [136,143,144]. RIPK1promotes astrogliosis and glial scar formation after ischemia stroke. The inhibition of RIPK1 can promote the restoration of brain function by inhibiting astrogliosis and glial scarring [137].

The RIPK1 kinase domain is an important disease-driver in a mouse model of ICH [138]. After ICH, increased interactions of RIPK1 and RIPK3, RIPK1 and MLKL, and RIPK1 and caspase-8 were observed in brain tissue. Additionally, the necroptosis pathway can be effectively blocked by knocking down RIP1 [145]. The protein and mRNA expression levels of RIPK1, RIPK3 and MLKL were increased 72 h after ICH. Nec-1 can improve brain edema and neuroinflammation by inhibiting necroptosis, and improve behavioral scores after ischemic brain injury [139,146] (Table 4).

### 8.3. Pyroptosis and Stroke

The classical pyroptotic pathway is mediated by caapase-1, and the role of caspase-1 in cerebral ischemia has been revealed, and knockout of caspase-1 or the use of caspase-1 inhibitors has a long-term neuroprotective effect on cerebral ischemic injury [147,148]. The assembly of the inflammasome is also a key part of the pyroptotic pathway during cerebral ischemia, and the upregulation of the NLRP3 inflammasome complex, as well as IL-1β and IL-18, has been demonstrated in the brain tissue of patients with cerebral ischemia [149]. Nervous system deterioration after cerebral ischemia can be prevented by inhibiting the expression of NLRP3 [150]. GSDMD, a key response component of pyroptosis, appeared at 12 h after cerebral ischemia and reached a peak at 24 h, which mainly occurred in microglia. Knockout of GSDMS can effectively reduce the infarct volume and block the release of mature IL-1β and IL-18, but does not affect the maturation process [140].

Increased GSDMD-positive microglia, increased GSDMD-N protein levels and IL-1β production were observed in the cerebral cortex in a mouse SAH model. Additionally, the pores formed by GSDMD-N in the microglia membrane were detected by transmission electron microscopy. The results show that the ICH model can induce microglial pyroptosis [141]. The NLRP3 protein was upregulated after ICH and SAH models and peaked at 24 h, accompanied by an elevation of the inflammatory cytokines IL-1β and IL-18 [151,152,153,154]. Meanwhile, a significant upregulation of caspase-1 was observed at 3 h after ICH, peaking at 24–72 h [152,153]. Lastly, by inhibiting, caspase 1 can significantly improve neurological damage and brain edema after ICH [155,156] (Table 4).

## 9. Conclusions

With the study of a series of emerging forms of programmed cell death, such as necroptosis, pyroptosis, and ferroptosis, these forms of cell death have their own important functions and roles in their respective environments. Exploring its specific mechanisms in various diseases, constantly provides new treatment options for clinical disease models. In the nervous system, the balance between life and death of cells plays a crucial role in the normal development of the nervous system and the occurrence and development of nervous system diseases. The pathogenesis of nervous system diseases is often very complex and the development of the disease is the result of a combination of factors. Three kinds of programmed death are implicated in the pathogenesis of various neurological diseases, and the corresponding channel inhibitors have a positive effect on delaying the development of the disease and improving the prognosis of patients. Whether the relationship between the three is synergistic or antagonistic remains unclear. We still need to further explore and understand the specific signaling and interaction of various programmed cell death in various diseases, in order to provide guidance for the prognosis and treatment of diseases.

## Figures and Tables

**Table 1:** The features of ferroptosis, necroptosis, and pyroptosis, apoptosis, and necrosis.

Cell Death	Morphological Features	Biochemical Features	Key Genes	Ref.
Ferroptosis	Intact cell membrane, normal nuclear morphology, atrophy of mitochondria, increased density of mitochondrial membrane, and reduced or disappeared density of mitochondrial cristae	Iron accumulation and lipid peroxidation	GPX4, SLC7A11, TFR1, Nrf2, NCOA4, ACSL4, FSP1	[6,7]
Necroptosis	Organelle swelling, loss of plasma membrane integrity, and the breakdown of cell membranes leads to the release of cell contents	RIPK1, RIPK3, MLKL phosphorylation and ubiquitination, and formation of necrotic complexes in the cytoplasm	RIPK1, RIPK3, MLKL	[8,9,10,11,12,13]
Pyroptosis	Cell swelling and formation of pores in the plasma membrane	Inflammasome formation, caspase and gasdermin activation and the release of numerous pro-inflammatory factors	caspase-1, GSDMD	[14,15,16,17]
Apoptosis	Nucleus fragmentation, plasma membrane blistering, cell contraction	The formation of apoptotic bodies	Caspase, Bcl-2, Bax, Fas	[18]

**Table 2:** Mechanisms of ferroptosis, necroptosis, and pyroptosis in neurodegenerative diseases.

Cell Death	Target/Compound	Model	Effect	Mechanism	Ref.
Ferroptosis	GSH	PD patients	Induction	Decreased GSH, resulting in the formation of toxic hydroxyl radicals and ROS.	[52]
	Deferiprone	PD patients	Inhibition	Using deferiprone to inhibit the accumulation of iron ions, the patient showed clinical and radiological improvement.	[53]
	Ferritin	AD patients	Induction	Increased ferritin in AD brains induces ferroptosis by enhancing oxidative stress through the fenton response.	[54]
	GSH	AD patients	Induction	Decreased GSH in hippocampus and frontal cortex is associated with decline in cognitive function.	[55]
	GPX4	Gpx4BIKO mice	Induction	Gpx4BIKO mice induce elevated lipid peroxidation leading to cognitive impairment and neurodegeneration.	[56]
	iron	HD patients	Induction	Altered iron homeostasis in the brain maybe involved in Huntington’s disease pathophysiology.	[57]
	Nrf2	HD mice and HD patients	Inhibition	The selective Nrf2 inducer MIND4–17 inhibits the expression of proinflammatory cytokines in primary microglia and astrocytes.	[58]
Necroptosis	RIPK1	PD mice	Induction	Necroptosis is involved in neurodegeneration of dopaminergic neurons through miR-425-mediated activation of RIPK1.	[59]
	Nec-1s	PD mice	Inhibition	Nec-1s inhibition of necroptosis effectively reduces DA neuron loss caused by MPTP-dependent mitochondrial intoxication.	[60]
	RIPK1, RIPK3 and MLKL	PD patients and PD mice	Induction	Key components of necroptosis mechanisms are activated in the axons and soma of dopaminergic neurons of SNpc in the PD model.	[61]
	granulovacuolar degeneration	AD patients	Induction	The presence of activated necrosome complexes in granulovacuolar degeneration and its association with neuronal loss.	[62]
	O-GlcNAcylation	AD patients and AD mice	Inhibition	O-linked β-N-acetylglucosaminylation (O-GlcNAcylation) ameliorates model neuronal death and cognitive dysfunction by inhibiting necroptosis.	[63]
	TNF/TNFR1	AD patients	Induction	TNF/TNFR1 mediates necroptosis in AD patients, resulting in neuronal loss.	[64]
	Nec-1	APP/PS1 double-transgenic mice	Inhibition	Nec-1 directly targets Aβ and tau proteins, attenuates brain cell death and improves cognitive impairment in AD models.	[65]
	Nec-1	R6/2 transgenic mice	Inhibition	Nec-1 suppresses necroptosis in R6/2 transgenic mice, thereby improving HD symptoms.	[66]
Pyroptosis	miR-7	PD patients and PD mice	Inhibition	Transfection of miR-7 inhibited the activation of the NLRP3 inflammasome in microglial cells, accompanied by an inhibition of caspase-1 activation and reduced IL-1β production.	[67]
	α-synuclein	PD patients	Induction	IL-1β, NLRP3 levels were significantly increased in PD. We also observed a linear correlation of NLRP3 with α-synuclein.	[68]
	Aβ	APP/PS1 double-transgenic mice	Induction	Aβ aggregates can activate the NLRP3 inflammatory pathway, and NLRP3 promotes the maturation of caspase-1.	[69]
	Aβ	APP/PS1 double-transgenic mice	Induction	Aβ regulates pyroptosis through NLRP3-caspase-1 signaling.	[70]
	GSDMD	AD patients	Induction	The increased expression of GSDMD in the cerebrospinal fluid of AD patients has certain diagnostic value for AD.	[71]
	NLRP3	R6/2 transgenic mice	Induction	NLRP3-mediated pyroptosis leads to degeneration of HD neurons.	[72]
	MCC950	R6/2 transgenic mice	Inhibition	MCC950 delays HD disease progression by inhibiting NLRP3.	[73]

**Table 3:** Mechanisms of ferroptosis, necroptosis, and pyroptosis in traumatic brain injury.

Cell Death	Target/Compound	Model	Effect	Mechanism	Ref.
Ferroptosis	fer-1	TBI mice	Inhibition	Fer-1 treatment reduces neuronal cell death and improves long-term cognitive and motor function.	[109]
	*EAAC1*	TBI male mice from *EAAC1* ^−/−^ colony	Induction	*EAAC1 ^−/−^* mice promoted increased ROS and neuronal damage after TBI model by inhibiting cysteine uptake.	[113]
Necroptosis	CHMP4B	TBI mice	Inhibition	CHMP4B improves motor and memory function in TBI model mice by alleviating necroptosis of microglia.	[114]
	RIPK1, RIPK3	TBI mice	Induction	Ripk3 global knockout animals, as well as neuronal RIPK1-deficient mice, were protected from chronic brain injury by inhibiting downstream pMLKL and improved neurocognitive function after TBI.	[115]
	RIPK3	TBI mice	Induction	RIPK3 was highly induced after TBI, and RIPK3 knockout reduced inflammation by inactivating the NLRP3 and NF-κB pathways, and attenuated brain injury after TBI.	[116]
Pyroptosis	AC-FLTD-CMK	TBI mice	Inhibition	AC-FLTD-CMK administration reduced a key protein of necroptosis in a TBI model, and attenuated neuronal damage and brain edema.	[117]
	VX765	TBI mice	Inhibition	VX765 provides neuroprotection in TBI model mice by inhibiting caspase-1.	[118]
	MCC950	TBI mice	Inhibition	MCC950 attenuated pyroptosis-induced inflammatory damage by inhibiting NLRP3 and significantly improved neurological function in TBI mice.	[119]

**Table 4:** Mechanisms of ferroptosis, necroptosis, and pyroptosis in stroke.

Cell Death	Target/Compound	Model	Effect	Mechanism	Ref.
Ferroptosis	iron	MCAO mice	Induction	Iron overload leads to increased brain edema and hemorrhage area in mice with cerebral ischemia through fenton translation.	[130]
	tempol	MCAO mice	Inhibition	In mice model of MCAO, tempol reversed the size of the infarct size increased by iron treatment.	[132]
	fer-1	ICH mice	Inhibition	The use of Fer-1 in ICH model mice can reduce neuronal death and improve neural function by inhibiting lipid ROS.	[135]
	GPX4	ICH rats	Inhibition	Overexpression of GPX4 ameliorated secondary brain injury after ICH by inhibiting ferroptosis.	[134]
Necroptosis	Nec-1	MCAO rats	Inhibition	Nec-1 inhibits necroptosis by inhibiting RIPK1 phosphorylation in rat model of MCAO.	[136]
	RIPK1	MCAO rats	Induction	A key regulator of necroptosis, RIPK1, is involved in astrogliosis and glial scar formation.	[137]
	RIPK1	ICH mice	Induction	RIPK1 leads to increased blood-brain barrier permeability and brain edema in ICH model mice through MLKL-mediated necroptosis.	[138]
	Nec-1	ICH mice	Inhibition	Nec-1 improves neurobehavioral ability and brain edema by inhibiting RIPK1/RIPK3 pathway after ICH in mice.	[139]
Pyroptosis	GSDMD	MCAO mice	Induction	GSDMD-induced pyroptosis leads to neurological deficits and abundant neuronal cell death in MCAO mice.	[140]
	TREM-1	SAH mice	Induction	TREM-1 exacerbates neuroinflammation through NLRP3 inflammasome-mediated pyroptosis after SAH.	[141]

## Data Availability

Not applicable.
